# Pharmacological Strategies for Providing Patients With Delirium Relief From Terminal Dyspnea: A Secondary Data Analysis

**DOI:** 10.1002/cam4.70677

**Published:** 2025-02-11

**Authors:** Takaaki Hasegawa, Masanori Mori, Takashi Yamaguchi, Kengo Imai, Yoshinobu Matsuda, Isseki Maeda, Yutaka Hatano, Naosuke Yokomichi, Jun Hamano, Tatsuya Morita, East Asian Collaborative Cross‐Cultural Study to Elucidate the Dying Process (EASED) investigators

**Affiliations:** ^1^ Center for Psycho‐Oncology and Palliative Care Nagoya City University Hospital Nagoya Japan; ^2^ Palliative and Supportive Care Division Seirei Mikatahara General Hospital Hamamatsu Japan; ^3^ Department of Palliative Medicine Kobe University Graduate School of Medicine Kobe Japan; ^4^ Seirei Hospice Seirei Mikatahara General Hospital Hamamatsu Japan; ^5^ Department of Psychosomatic Internal Medicine NHO Kinki Chuo Chest Medical Center Sakai Japan; ^6^ Department of Palliative Care Senri‐Chuo Hospital Toyonaka Japan; ^7^ Department of Palliative Care Daini Kyoritsu Hospital Kawanishi Japan; ^8^ Department of Palliative and Supportive Care University of Tsukuba Tsukuba Japan

**Keywords:** antipsychotic agents, delirium, dyspnea, neoplasm, opioid, palliative care

## Abstract

**Introduction:**

Systemic opioids are recommended as a pharmacological treatment for dyspnea, and antipsychotics are widely used for delirium. Because little is known about optimal palliative pharmacological strategies for dyspnea in patients with delirium, this study explored the symptom course in such cases, including the use of opioids and antipsychotics.

**Methods:**

This was a secondary analysis of a multicenter prospective and observational study. We consecutively enrolled adult patients with advanced cancer at palliative care units in Japan. The eligibility criteria for their participation were a dyspnea Integrated Palliative care Outcome Scale (IPOS) score ≥ 2 and the presence of delirium. We investigated pharmacological strategies, IPOS for dyspnea, and delirium symptoms using item 9 of the Memorial Delirium Assessment Scale.

**Results:**

Of the 1896 patients, 141 were found eligible and were analyzed. Eighty‐two (58%) patients had agitated delirium, and the median survival period was 4 days. Regarding pharmacological strategy, 31 (22%) received opioid initiation or dose escalation, whereas 92 (65%) used regular antipsychotics. Although mean dyspnea IPOS scores significantly decreased from Day 1 to Day 2 (0.44, 95% CI: 0.24–0.64), the proportion of responders (IPOS score ≤ 1) was 21% (30/141). In the agitated delirium group, the proportion of remaining agitation symptoms at Day 2 was 74% (61/82).

**Conclusions:**

The combined distressing symptoms of dyspnea and delirium during the last days of life are likely to be refractory suffering, which shows a poor response to pharmacological interventions, including opioids and antipsychotics.

## 1 Introduction

Dyspnea is common, and manifests distressing symptoms in patients with advanced cancer [1]. Opioid is the first‐line palliative pharmacological therapy for dyspnea [2, 3, 4, 5], and its effectiveness has been reported in prospective observational studies for terminal dyspnea [6, 7]. However, opioids can cause delirium or exacerbate the symptoms of delirium [8, 9]. Hence, opioids should be used with caution in the treatment of dyspnea with delirium. Although antipsychotics themselves are not expected to relieve dyspnea, as the delirium itself can exacerbate dyspnea [10], administration of antipsychotics combined with opioids is considered a reasonable option for dyspnea with delirium.

Nonetheless, little is known about optimal palliative pharmacological strategies, including opioids and antipsychotics for dyspnea, in patients with delirium [7]. In addition, Japanese guidelines (for dyspnea and delirium) do not provide any recommended pharmacotherapy for dyspnea complicated by delirium in patients with cancer [4, 11]. Therefore, this study explored the real‐world symptom course of pharmacological strategies, including opioids and antipsychotics, for dyspnea with delirium among patients on the verge of dying. Additionally, it examined changes in delirium symptoms using these strategies.

## 2 Methods

This study is a secondary analysis of a longitudinal study focusing on the dying process and end‐of‐life care in multicenter inpatient hospices or palliative care units, which had been previously reported [12]. While the details of this study—East‐Asian collaborative cross‐cultural study to elucidate the dying process (EASED)—have been described elsewhere, a brief summary is that we prospectively studied patients with terminal cancer at multicenter establishments in Japan. The institutional review board at each site approved the study's protocol. The study was conducted in accordance with the principles of the Declaration of Helsinki. The ethical guidelines for epidemiological research presented by the Ministry of Health, Labor, and Welfare in Japan do not require investigators to obtain the informed consent of participants in a non‐invasive observational study such as this one [13]. Therefore, rather than acquiring written or oral informed consent, we used an opt‐out method wherein all patients could receive information on the study through instructions posted on the ward or institutional website, and they had the opportunity to decline participation.

### 2.1 Participants

Patients were included for participation in this study if they were: (1) diagnosed with locally advanced or metastatic cancer, (2) aged ≥ 18 years, and (3) admitted to an inpatient hospice or palliative care unit. They were excluded if they were scheduled for discharge in ≤ 1 week, or had declined to participate in this study. For this secondary analysis, the inclusion criteria were as follows: (a) those with a Palliative Performance Scale (PPS) score ≤ 20 (i.e., totally bed bound, requiring full assistance and minimal oral intake limited to sips or mouth care only), (b) those having an Integrated Palliative Outcome Scale (IPOS) score for dyspnea ≥ 2 at the time of showing a PPS score ≤ 20, and (c) those who had been diagnosed with delirium by palliative care physicians based on the 5th edition of the Diagnostic and Statistical Manual of Mental Disorders (DSM‐5). The exclusion criteria were: (a) discharged alive without developing a PPS score ≤ 20, (b) showed a PPS score ≤ 20 but died on the same day, or (c) had no available data of pharmacological treatment (relating to using or not using opioids and antipsychotics).

### 2.2 Measurement

#### 2.2.1 Pharmacological Strategy

We evaluated regular use of any opioid and its oral morphine equivalent daily dose at Day 1 (at the point of showing a PPS score ≤ 20), and those between Day 1 and the next day (Day 2). We also recorded the use of antipsychotics at Day 1. From Day 1 to Day 2, newly initiating opioids for opioid‐naïve patients or dose escalation from baseline opioid dose (any type of opioid) were defined as opioid initiation or dose escalation. The pharmacological strategy of opioid and antipsychotics were classified as follows: “no opioid initiation or dose escalation without antipsychotics,” “opioid initiation or dose escalation without antipsychotics,” “no opioid initiation or dose escalation with antipsychotics” and “opioid initiation or dose escalation with antipsychotics.”

#### 2.2.2 Dyspnea

The staff proxy version of the IPOS for dyspnea was used to evaluate the severity of dyspnea symptoms. This version is a clinician‐rated scale for symptom assessment, and its scores range from 0 to 4 (0 = not at all, 1 = slightly, 2 = moderately, 3 = severely, 4 = overwhelmingly) [14, 15]. Cases in which the IPOS score for dyspnea could not be assessed owing to the loss of consciousness (e.g., initiation of sedation or the natural dying process) were marked as “Cannot assess”. IPOS, a validated scale for assessing dyspnea in patients receiving palliative care, is useful for assessing dyspnea, especially in patients who cannot rate or provide self‐evaluation reports [16].

#### 2.2.3 Delirium

As mentioned, delirium was diagnosed by palliative care physicians using DSM‐5 on Day 1 (at the point of showing a PPS score ≤ 20). Its subtypes (hyperactive or mixed type, and hypoactive delirium) were also simultaneously assessed. Item 9 of the Memorial Delirium Assessment Scale (MDAS) was used to assess the severity of agitated delirium, rated 0 (normal)–3 (severe) [17]. Item 4 of the Communication Capacity Scale (CCS) was used to assess the maintenance of communication capacity, rated 0–3 [18]. Lower scores indicate that communication capacity is maintained. The modified Richmond Agitation‐Sedation Scale (RASS) was used to assess the severity of agitation and sedation levels [19, 20, 21].

#### 2.2.4 Palliative Sedation (Intermittent or Continuous Sedation)

Continuous sedation, defined as “continuous infusion of a sedative for the purpose of alleviating refractory symptoms with the intention to keep a patient continuously unconscious,” and intermittent sedation were also recorded. In this study, the sedative included only midazolam.

### 2.3 Procedure

Consecutive patients in inpatient hospices or palliative care units, who had an IPOS score for dyspnea ≥ 2 at the time of showing a PPS score ≤ 20 and delirium, were enrolled in this secondary analysis. In this study, the point of PPS ≤ 20 was defined as occurring at the time immediately before death. A total of 23 inpatient hospices or palliative care units participated in this study. The patients were enrolled between January 2017 and December 2017. End‐of‐life care, including treatment and evaluation of outcomes, including symptoms (e.g., dyspnea and delirium), were conducted within routine clinical practice.

All data for dyspnea and delirium, including IPOS for dyspnea (worst over 24 h), respiratory rate, MDAS item 9 (worst over 24 h), CCS item 4 (best over 24 h), and RASS score were assessed by the responsible physicians on Day 1 (at the point of showing a PPS score ≤ 20), and on Day 2.

Upon admission to an inpatient hospice or palliative care unit, the following details were obtained from medical records as baseline data: age, sex, primary cancer site, metastatic lesions, comorbidity, and smoking history. At Day 1 (the point showing a PPS score ≤ 20), IPOS scores for pain, use of supplemental oxygen, and regular use of corticosteroids were also assessed.

### 2.4 Statistical Analyses

We commenced analyses with descriptive summaries of demographic and clinical variables. As this secondary and exploratory analysis mainly focused on providing relief from dyspnea and changes in its intensity based on each pharmacological strategy, we assessed relief from dyspnea using the proportion of responders at Day 2. We defined responders as those having a ≤ 1 IPOS score for dyspnea [6, 22] at Day 2, which signifies no dyspnea or mild or occasional dyspnea (i.e., The patient is not bothered by dyspnea). This is clinically considered satisfactory dyspnea relief [7]. We also assessed changes in dyspnea intensity between Days 1 and 2 using paired *t*‐test. As this cohort included patients immediately before death, we assumed that the initiation of palliative sedation or the natural dying process would frequently result in missing IPOS values (i.e., “Cannot assess” cases) at Day 2. Therefore, we performed paired *t*‐test using complete‐case and sensitivity analyses for missing data through baseline observation carried forward analysis.

For our secondary focus areas—delirium symptoms and adverse effects of each pharmacological strategy—we used descriptive analyses or paired *t*‐test between Days 1 and 2 when appropriate. We also conducted these analyses among subgroups of patients with different delirium motor subtypes (hyperactive or mixed, or hypoactive delirium). We conducted these analyses using SPSS v.28.0 (IBM Corp., Armonk, NY, USA). Statistical significance was set at *p* < 0.05.

## 3 Results

**FIGURE 1:**
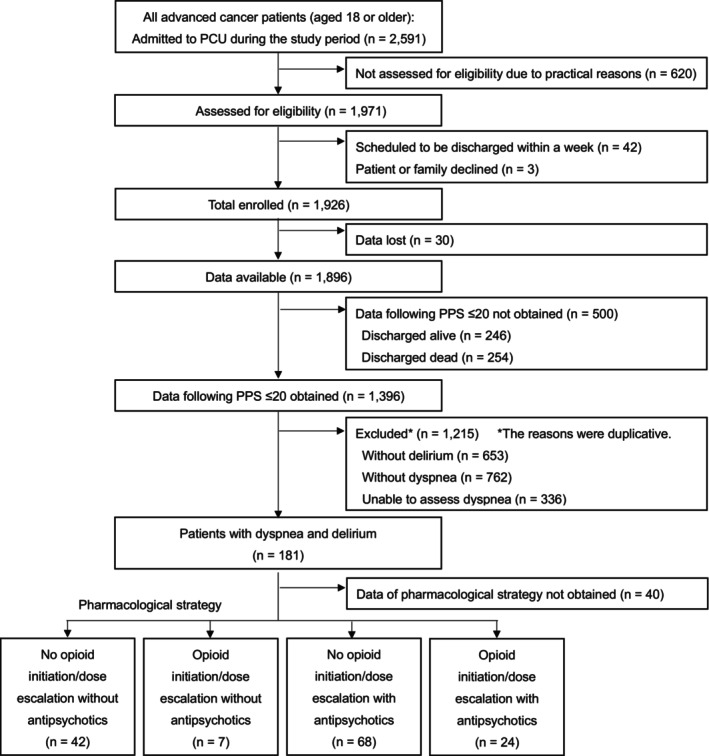
Flow chart showing the recruitment of participants. PCU, palliative care unit; PPS, palliative performance scale.

Figure 1 shows the flow diagram for the recruitment of participants. A total of 141 eligible patients enrolled in this study. Table 1 shows the patients' characteristics. The patients' mean age was 72 (standard deviation: 12.6); 42% were female; the most common primary cancer site was the lungs (33%); 42% had lung metastasis; 86% received supplemental oxygen therapy; 36% had regular use of corticosteroids, and 90% were opioid‐tolerant. The median survival period was 4 days (interquartile range: 3–8).

**TABLE 1:** Patients' characteristics.

	Total (*n* = 141)	No opioid initiation or dose escalation without antipsychotics (*n* = 42)	Opioid initiation or dose escalation without antipsychotics (*n* = 7)	No opioid initiation or dose escalation with antipsychotics (*n* = 68)	Opioid initiation or dose escalation with antipsychotics (*n* = 24)	
	*n* (%)	*n* (%)	*n* (%)	*n* (%)	*n* (%)	*p*
Age, years						0.65
Mean (SD)	72 (12.6)	74 (14.4)	74 (12.0)	71 (11.2)	70 (13.5)	
Sex						0.13
Female	59 (42)	24 (57)	2 (29)	24 (35)	9 (38)	
Male	82 (58)	18 (43)	5 (71)	44 (65)	15 (63)	
Primary cancer site						0.13
Lung	47 (33)	11 (26)	1 (14)	27 (40)	8 (33)	
Gastrointestinal	30 (21)	13 (31)	5 (71)	10 (15)	2 (8)	
Genitourinary	10 (7)	1 (2)	0 (0)	7 (10)	2 (8)	
Liver, bile duct	9 (6)	4 (9)	0 (0)	5 (7)	0 (0)	
Pancreas	7 (5)	3 (7)	0 (0)	4 (6)	0 (0)	
Gynecological	7 (5)	2 (5)	1 (14)	2 (3)	2 (8)	
Head and neck	7 (5)	3 (7)	0 (0)	4 (6)	0 (0)	
Other	24 (17)	5 (12)	0 (0)	9 (13)	10 (41)	
Metastatic lesions						
Lung metastasis	59 (42)	14 (33)	6 (86)	27 (40)	12 (50)	0.59
Liver metastasis	43 (31)	11 (26)	3 (43)	18 (27)	11 (46)	0.24
Brain metastasis	21 (15)	3 (7)	0 (0)	13 (19)	5 (21)	0.20
Comorbidity						
Diabetes	14 (10)	4 (10)	1 (14)	5 (7)	4 (17)	0.45
Chronic lung disease	13 (9)	4 (10)	0 (0)	4 (6)	5 (21)	0.17
Cerebral vascular disease	11 (8)	3 (7)	1 (14)	6 (9)	1 (4)	0.71
Cognitive impairment	10 (7)	3 (7)	0 (0)	5 (7)	2 (8)	1.00
Congestive heart failure	7 (5)	2 (5)	0 (0)	4 (6)	1 (4)	1.00
Smoking history						0.37
Current	6 (4)	1 (2)	1 (14)	4 (6)	0 (0)	
Former	55 (39)	14 (33)	3 (43)	25 (37)	13 (54)	
Never	45 (32)	15 (36)	1 (14)	21 (31)	8 (33)	
Unknown	35 (25)	12 (29)	2 (29)	18 (27)	3 (13)	
Pain (IPOS)						0.69
Mean (SD)	1.2 (1.0)	1.0 (1.2)	1.4 (1.4)	1.2 (0.9)	1.3 (1.0)	
Supplemental oxygen						0.90
Yes	121 (86)	36 (86)	7 (100)	57 (84)	21 (88)	
No	20 (14)	6 (14)	0 (0)	11 (16)	3 (13)	
SpO2, %						0.033
Mean (SD)	94.5 (4.3)	95.0 (3.9)	90.7 (8.9)	94.1 (4.1)	95.8 (2.7)	
Regular use of corticosteroid						0.47
Yes	51 (36)	19 (45)	2 (29)	21 (31)	9 (38)	
No	90 (64)	23 (55)	5 (71)	47 (69)	15 (63)	
Subtypes of delirium						0.21
Hyperactive or mixed	82 (58)	20 (48)	3 (43)	42 (62)	17 (71)	
Hypoactive	59 (42)	22 (52)	4 (57)	26 (38)	7 (29)	
Regular prior use of opioid						0.91
Yes	127 (90)	38 (91)	6 (86)	61 (90)	22 (92)	
No	14 (10)	4 (10)	1 (14)	7 (10)	2 (8)	
Prior opioid dose among patients on regular opioids	(*n* = 127)	(*n* = 38)	(*n* = 6)	(*n* = 61)	(*n* = 22)	
Mean (SD)	107 (157)	100 (193)	104 (160)	118 (156)	90 (82)	0.78
Median (IQR)	72 (24, 96)	44 (20, 84)	45 (12, 173)	72 (32, 120)	82 (29, 96)	0.16

Abbreviations: IPOS, integrated palliative outcome scale; IQR, interquartile range; SD, standard deviation.

Regarding pharmacological strategies, “no opioid initiation or dose escalation with antipsychotics” was used in 68 (48%) patients, followed by “no opioid initiation or dose escalation without antipsychotics” in 42 (30%) patients, “opioid initiation or dose escalation with antipsychotics” in 24 (17%) and “opioid initiation or dose escalation without antipsychotics” in 7 (5%) patients.

### 3.1 Change in Dyspnea Intensity by Pharmacological Strategy

**FIGURE 2:**
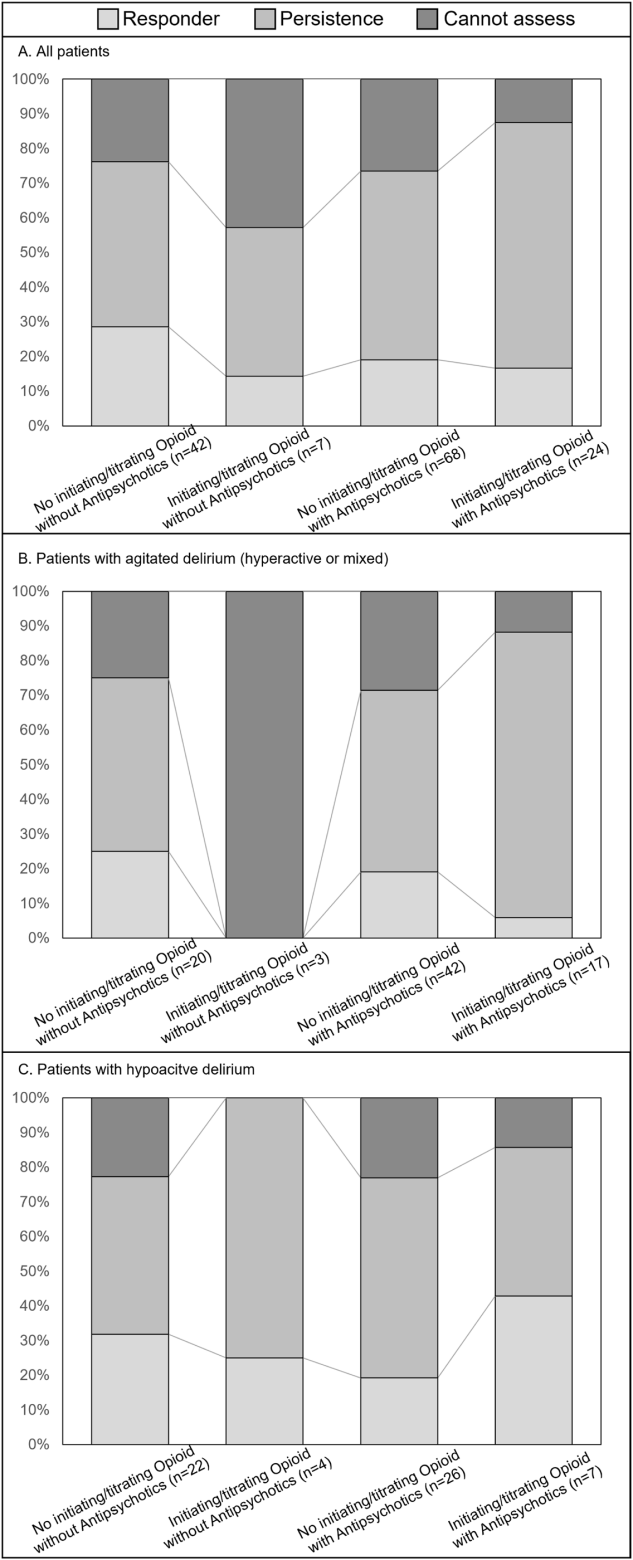
Proportion of patients based on longitudinal changes in dyspnea intensity (IPOS score for dyspnea) at Day 2. Responder means having an IPOS score for dyspnea ≤ 1. Persistence means an IPOS score for dyspnea ≥ 2. “Cannot assess” signifies no available responses owing to an IPOS score for dyspnea at day 2 categorized as “Cannot assess.” IPOS, integrated palliative outcome scale.

Figure 2, Tables S1 and S2 show the proportion of responders: 29% (12/42, 95% confidence interval [CI]: 17–44) in the “no opioid initiation or dose escalation without antipsychotics” group, 14% (1/7, 95% CI: 1–44) in the “opioid initiation or dose escalation without antipsychotics” group, 19% (13/68, 95% CI: 11–33) in the “no opioid initiation or dose escalation with antipsychotics” group, and 17% (4/24, 95% CI: 6–37) in the “opioid initiation or dose escalation with antipsychotics” group.

**FIGURE 3:**
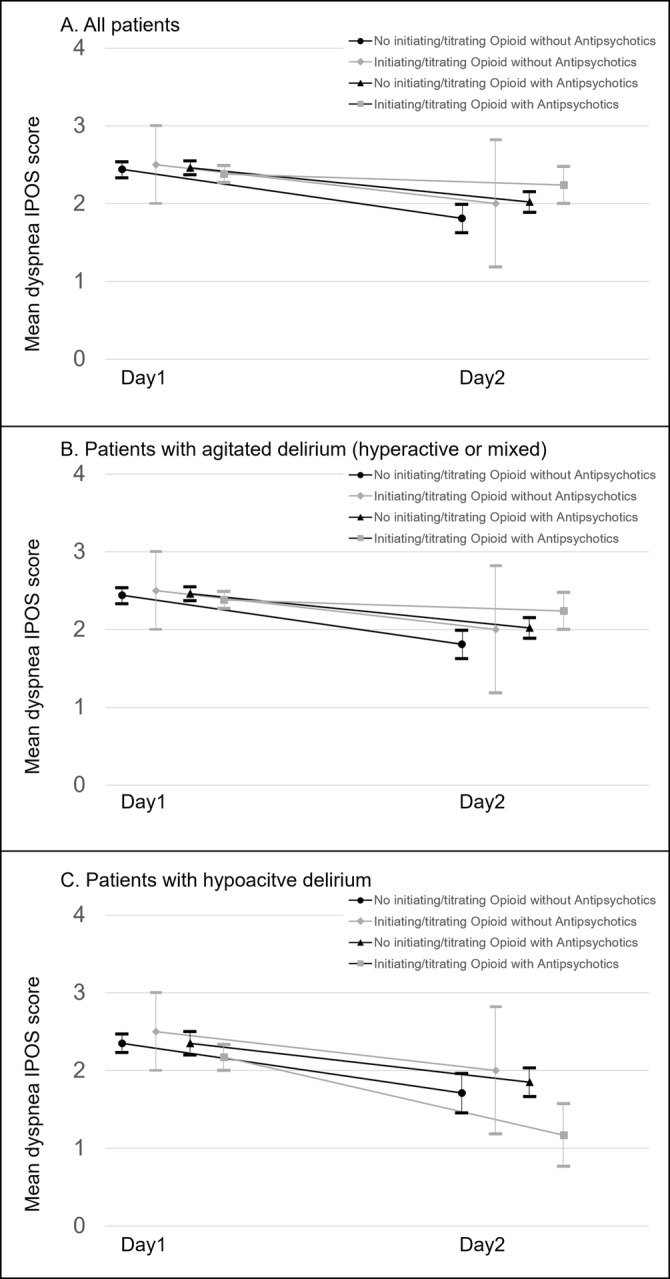
Changes in the mean scores of IPOS for dyspnea. Bars show standard error. IPOS, integrated palliative outcome scale.

Of the 141 participants, an IPOS rating for dyspnea was missing in 34 cases (i.e., “Cannot assess”). In terms of dyspnea intensity, the complete case analysis showed a decrease in the mean IPOS score for dyspnea (Figure 3 and Table S3). The “no opioid initiation or dose escalation without antipsychotics” and “no opioid initiation or dose escalation with antipsychotics” groups achieved a statistically significant difference, but the “opioid initiation or dose escalation without antipsychotics” and “opioid initiation or dose escalation with antipsychotics” groups did not. In the subgroup of patients with agitated delirium (hyperactive or mixed) receiving opioid initiation or dose escalation with or without antipsychotics, the mean IPOS score for dyspnea either increased or did not change. We also performed sensitivity analyses using the baseline observation carried forward method, and the same results were reproduced (Table S4).

No significant changes in respiratory rate were observed in all the pharmacological strategy groups (Table 2).

**TABLE 2:** Change in outcomes (mean scores) other than dyspnea intensity.

Complete case analysis among all patients (*n* = 141^a^)																
	No opioid initiation or dose escalation without antipsychotics				Opioid initiation or dose escalation without antipsychotics				No opioid initiation or dose escalation with antipsychotics				Opioid initiation or dose escalation with antipsychotics			
	Day 1	Day 2 (SD)	Difference	*p*	Day 1	Day 2	Difference	*p*	Day 1	Day 2	Difference	*p*	Day 1	Day 2	Difference	*p*
	(SD)		(95% CI)		(SD)	(SD)	(95% CI)		(SD)	(SD)	(95% CI)		(SD)	(SD)	(95% CI)	
MDAS	0.9	0.5	0.4 (0.1–0.7)	0.01	0.6	0.1	0.4 (−0.3–1.2)	0.20	1.1	0.7	0.3 (0.1–0.5)	0.001	0.8	0.8	0.08 (−0.2–0.3)	0.49
Item 9	(1.0)	(0.7)			(0.8)	(0.4)			(0.9)	(0.8)			(0.7)	(0.8)		
CCS	1.7	2.1	−0.5 (−0.7 to −0.2)	< 0.001	2.1	2.6	−0.4 (−0.9–0.1)	0.078	1.8	2.2	−0.4 (−0.5 to −0.2)	< 0.001	1.5	1.9	−0.4 (−0.7 to −0.0)	0.036
Item 4	(0.9)	(0.9)			(0.7)	(0.8)			(0.8)	(0.9)			(0.8)	(0.8)		
RASS	−0.4	−1.8	1.4 (0.8–2.0)	< 0.001	−1.9	−2.9	1.0 (0.5–1.5)	0.004	−0.9	−1.9	1.0 (0.6–1.4)	< 0.001	−0.5	−1.5	1.0 (0.5–1.5)	< 0.001
	(1.6)	(1.7)			(0.7)	(0.7)			(1.4)	(1.6)			(1.0)	(1.3)		
RR	16.6	15.8	1.2 (−1.0–3.4)	0.28	20.3	16.9	3.4 (−2.9–9.7)	0.23	15.7	15.3	0.1 (−1.5–1.7)	0.87	17.0	18.2	−0.8 (−4.4–2.9)	0.66
	(7.5)	(5.7)			(6.5)	(4.5)			(5.2)	(5.0)			(6.1)	(6.2)		

Complete case analysis among patients with agitated delirium (hyperactive or mixed; *n* = 82^b^)																
	No opioid initiation or dose escalation without antipsychotics				Opioid initiation or dose escalation without antipsychotics				No opioid initiation or dose escalation with antipsychotics				Opioid initiation or dose escalation with antipsychotics			
	Day 1	Day 2	Difference	*p*	Day 1	Day 2	Difference	*p*	Day 1	Day 2	Difference	*p*	Day 1	Day 2	Difference	*p*
	(SD)	(SD)	(95% CI)		(SD)	(SD)	(95% CI)		(SD)	(SD)	(95% CI)		(SD)	(SD)	(95% CI)	
MDAS	1.6	0.8	0.8 (0.3–1.3)	0.006	1.3	0.3	1.0 (−1.5–3.5)	0.23	1.7	1.1	0.6 (0.2–0.9)	0.001	1.2	1.1	0.1 (−0.2–0.5)	0.50
Item 9	(0.8)	(0.8)			(0.6)	(0.6)			(0.7)	(0.8)			(0.4)	(0.7)		
CCS	1.7	2.1	−0.5 (−0.7 to −0.2)	0.004	2.0	3.0	NA	NA	2.0	3.0	NA	NA	1.6	1.9	−0.4 (−0.8–0.1)	0.11
Item 4	(1.0)	(1.0)			(0)	(0)			(0)	(0)			(0.7)	(0.7)		
RASS	0.7	−1.3	2.0 (0.9–3.1)	0.001	−1.7	−3.0	1.3 (−0.1–2.8)	0.057	−0.7	−1.9	1.2 (0.6–1.7)	< 0.001	−0.4	−1.5	1.1 (0.6–1.7)	< 0.001
	(1.7)	(2.0)			(0.6)	(0)			(1.6)	(1.7)			(1.1)	(1.2)		
RR	14.5	15.0	0.2 (−3.4–3.9)	0.90	20.0	13.3	6.6 (−8.5–21.8)	0.20	16.2	15.1	0.8 (−1.6–3.2)	0.52	17.6	20.4	−2.4 (−7.1–2.3)	0.29
	(6.6)	(5.9)			(9.2)	(4.2)			(5.9)	(5.2)			(6.1)	(6.1)		

Complete case analysis among patients with hypoactive delirium (*n* = 59^c^)																
	No opioid initiation or dose escalation without antipsychotics				Opioid initiation or dose escalation without antipsychotics				No opioid initiation or dose escalation with antipsychotics				Opioid initiation or dose escalation with antipsychotics			
	Day 1	Day 2	Difference	*p*	Day 1	Day 2	Difference	*p*	Day 1	Day 2	Difference	*p*	Day 1	Day 2	Difference	*p*
	(SD)	(SD)	(95% CI)		(SD)	(SD)	(95% CI)		(SD)	(SD)	(95% CI)		(SD)	(SD)	(95% CI)	
MDAS	0.2	0.1	0.0 (−0.2–0.3)	0.75	0	0	NA	NA	0.1	0.1	NA	NA	0	0	NA	NA
Item 9	(0.5)	(0.5)			(0)	(0)			(0.3)	(0.3)			(0)	(0)		
CCS	1.7	2.2	−0.45 (−0.8 to −0.1)	0.009	2.3	2.3	NA	NA	1.5	1.8	−0.31 (−0.5 to −0.1)	0.008	1.3	1.7	−0.43 (−1.2–0.3)	0.20
Item 4	(0.9)	(0.9)			(1.0)	(1.0)			(0.8)	(1.0)			(1.0)	(1.1)		
RASS	−1.4	−2.3	0.91 (0.4–1.4)	0.002	−2.0	−2.8	0.8 (−0.0–1.5)	0.058	−1.1	−1.9	0.8 (0.2–1.4)	0.013	−0.9	−1.4	0.6 (−0.7–1.9)	0.32
	(0.7)	(1.1)			(0.8)	(1.0)			(0.8)	(1.4)			(0.7)	(1.6)		
RR	18.4	16.7	2.1 (−0.8–5.1)	0.13	20.5	19.5	1.0 (−10.3–12.3)	0.80	15.0	15.8	−0.8 (−2.7–1.1)	0.39	15.9	13.3	2.6 (−3.7–8.8)	0.35
	(7.9)	(5.6)			(5.2)	(2.6)			(3.9)	(4.9)			(6.5)	(2.9)		

Abbreviations: CCS, communication capacity scale; MDAS, memorial delirium assessment scale; NA, not available; RASS, modified Richmond agitation‐sedation scale; RR, respiratory rate; SD, standard deviation.

^a^ Denotes the number of missing data for RASS score and RR, which was 4 and 21, respectively.

^b^ Denotes the number of missing data for MDAS item 9 and RR, which was 1 and 14, respectively.

^c^ Denotes number of missing data for RASS score and RR, which was 1 and 7, respectively.

### 3.2 Changes in Delirium Symptoms by Pharmacological Strategy

At Day 1, 58% had agitated delirium (hyperactive or mixed), and the remaining 42% had hypoactive delirium (Table 1).

In terms of severity of agitation, the mean of MDAS item 9 decreased in three groups, but not for the “opioid initiation or dose escalation with antipsychotic” group (Tables 2 and S1). The “no opioid initiation or dose escalation without antipsychotics” and “no opioid initiation or dose escalation with antipsychotics” groups achieved a statistically significant difference. In the agitated delirium group, the proportion of remaining agitation symptoms was 74% (61/82).

In terms of communication capacity, CCS item 4 deteriorated among patients in all groups. Similarly, RASS revealed deepened sedation levels among patients in all groups (Table 2).

### 3.3 Frequency of Palliative Sedation (Intermittent or Continuous Sedation) in Each Pharmacological Strategy

The proportions of patients who received palliative sedation using midazolam are shown in Table 3. The proportions of patients who received intermittent and continuous sedation slightly varied. No participants received new initiation of palliative sedation after Day 2.

**TABLE 3:** Proportion of sedation.

All patients (*n* = 141)															
No opioid initiation or dose escalation without antipsychotics (*n* = 42)				Opioid initiation or dose escalation without antipsychotics (*n* = 7)				No opioid initiation or dose escalation with antipsychotics (*n* = 68)				Opioid initiation or dose escalation with antipsychotics (*n* = 24)			
Intermittent sedation		Continuous deep sedation		Intermittent sedation		Continuous deep sedation		Intermittent sedation		Continuous deep sedation		Intermittent sedation		Continuous deep sedation	
%	(95% CI)	%	(95% CI)	%	(95% CI)	%	(95% CI)	%	(95% CI)	%	(95% CI)	%	(95% CI)	%	(95% CI)
19	(10–34)	7	(2–20)	0	(0–40)	14	(1–53)	21	(13–32)	15	(8–25)	13	(4–32)	21	(9–41)

Patients with agitated delirium (hyperactive or mixed; *n* = 82)															
No opioid initiation or dose escalation without antipsychotics (*n* = 20)				Opioid initiation or dose escalation without antipsychotics (*n* = 3)				No opioid initiation or dose escalation with antipsychotics (*n* = 42)				Opioid initiation or dose escalation with antipsychotics (*n* = 17)			
Intermittent sedation		Continuous deep sedation		Intermittent sedation		Continuous deep sedation		Intermittent sedation		Continuous deep sedation		Intermittent sedation		Continuous deep sedation	
%	(95% CI)	%	(95% CI)	%	(95% CI)	%	(95% CI)	%	(95% CI)	%	(95% CI)	%	(95% CI)	%	(95% CI)
30	(14–52)	10	(2–31)	0	(0–62)	33	(6–80)	24	(13–39)	19	(10–33)	18	(5–42)	29	(13–53)

Patients with hypoactive delirium; *n* = 59															
No opioid initiation or dose escalation without antipsychotics (*n* = 22)				Opioid initiation or dose escalation without antipsychotics (*n* = 4)				No opioid initiation or dose escalation with antipsychotics (*n* = 26)				Opioid initiation or dose escalation with antipsychotics (*n* = 7)			
Intermittent sedation		Continuous deep sedation		Intermittent sedation		Continuous deep sedation		Intermittent sedation		Continuous deep sedation		Intermittent sedation		Continuous deep sedation	
%	(95% CI)	%	(95% CI)	%	(95% CI)	%	(95% CI)	%	(95% CI)	%	(95% CI)	%	(95% CI)	%	(95% CI)
9	(1–29)	5	(0–24)	0	(0–55)	0	(0–55)	15	(6–34)	8	(1–25)	0	(0–40)	0	(0–40)

Abbreviation: CI, confidence interval.

## 4 Discussion

This nationwide observational study showed the real‐world dyspnea course of several pharmacological strategies in patients with terminal delirium immediately before their death. In settings involving inpatient‐specialized palliative care services, the major practice for an opioid strategy to address terminal dyspnea complicated by delirium is that of no opioid initiation or dose escalation (78%: 110/141). Nevertheless, a constant tendency toward decreased communication capacity and a relatively high sedation rate were observed. These data might represent a situation wherein 90% of patients had already been introduced to opioids at the inception point, and when palliative care physicians diagnosed refractory dyspnea, omitted further opioid dose escalation, or when they were diagnosed with refractory dyspnea shortly after an opioid titration. That is, these data might represent records of terminally ill patients who were diagnosed with refractory dyspnea without sufficient time having elapsed after titrating opioids. The trend toward a reduced mean IPOS score for dyspnea suggests that some of the patients could have experienced relief from dyspnea. Alternatively, the physicians might have perceived the decreased level of consciousness during the natural dying process as a decrease in the intensity of dyspnea. Furthermore, there might have been a regression to the mean, owing to the selection of only patients with both dyspnea and delirium when the PPS score decreased to ≤ 20. These factors may explain why the results differ from those of previous studies, which indicate that dyspnea tends to worsen just before death [23, 24]. However, the changes in the mean IPOS scores for dyspnea in this study were smaller than those in previous studies for terminal dyspnea [6, 25].

A population of special note in this secondary data analysis was the one that received opioid initiation or dose escalation for terminal dyspnea with agitated delirium (hyperactive or mixed). This population's relief from dyspnea response rate (17 and 3 with and without antipsychotics, respectively) was remarkably low (5%: 1/20). Hence, caution is required when using the pharmacological strategy of opioid initiation or dose escalation for this population. Furthermore, the average dyspnea intensity tended to be worse in the subgroup with “opioid initiation or dose escalation with antipsychotics,” even though it was assumed that opioid initiation or dose titration would reduce dyspnea intensity, and antipsychotics would reduce symptoms of delirium, which in turn would modify symptoms of dyspnea. Development of a novel treatment strategy may be needed particularly for patients with both agitated delirium and terminal dyspnea.

The response rates of dyspnea relief for all four treatment strategies, with or without opioid initiation or dose escalation, and with or without antipsychotics, were generally lower (21%: 30/141) than those previously reported for terminal dyspnea [6, 25]. The level of consciousness decreased among a non‐negligible proportion of participants; dyspnea intensity became unevaluable (24%: 34/141), while more than half (54%: 77/141) had persistent dyspnea (IPOS score for dyspnea ≥ 2). Although this secondary analysis focused on two symptoms: “delirium and dyspnea,” it may have assessed complex refractory suffering because of the difficulty in distinguishing and assessing individual symptoms [26, 27, 28]. It is also assumed that individually managing a single symptom that constitutes complex intolerable suffering may be unsuccessful. Hence, it may be necessary to proactively discuss prioritizing or valuing the wishes of patients or caregivers, considering prospects for refractory or proportionality, and examining palliative sedation for alleviating complex suffering.

The preliminary findings of this exploratory secondary analysis have implications about terminal dyspnea complicating delirium. First, a seamless treatment strategy that includes opioids, antipsychotics, and sedatives, as well as relief algorithms for each symptom, must be developed. Second, prospective observational studies that consider the appropriate timing of assessment of terminal dyspnea complicating delirium (particularly hyperactive or mixed delirium) are needed. Currently, the appropriate timing for assessing terminal dyspnea complicated by delirium has not been established. Third, the appropriate outcomes in patients with terminal dyspnea that complicates delirium require further discussion. While “relief from dyspnea,” “reducing the severity of delirium symptoms,” and “maintaining communication capacity” would be ideal, due consideration must be given to the feasibility of a combined outcome and to setting priorities [21, 29]. Fourth, in the assessment of terminal dyspnea, when dyspnea intensity can no longer be assessed (not available owing to a reduced state of consciousness), whether the condition should be rated as good or bad must be discussed.

Regarding limitations, the present study has several that should be considered. First, it assessed dyspnea intensity as reported by physicians, not patients (e.g., numerical rating scale [NRS]). However, it managed to include patients who could not respond to the NRS by using the staff proxy version of the IPOS for dyspnea of terminally ill patients, one of the validated methods [16]. Second, as this study was a secondary analysis, it was not possible to examine the usefulness of antipsychotics. Moreover, detailed data on the type and dosage of antipsychotics was not obtained, as this analysis was not designed to allow examining group differences in each pharmacological strategy. Third, the intentions for using each pharmacological strategy were not assessed, although various factors, such as opioid initiation or dose escalation for pain management, might be involved. However, a few participants had severe pain, and the average IPOS for pain at Day 1 was 1.2. Fourth, co‐interventions, particularly non‐pharmacological treatments for dyspnea or delirium, were not recorded; hence, their impact on the intensity of dyspnea and delirium could not be assessed [30]. Fifth, the study did not document the rescue usage of opioids, antipsychotics, and benzodiazepines. The results suggest that opioid initiation or dose escalation is not recommended, but the rescue usage of opioids or antipsychotics should not be ruled out. Sixth, the symptom course for opioid initiation, rather than opioid dose escalation, could not be evaluated because 90% (127/141) of the participants were patients who were already regular opioid users. Seventh, this study was conducted in settings of inpatient‐specialized palliative care services. Hence, its findings cannot be generalized to other settings, such as non‐specialized palliative care services, other clinical stages of cancer, or preserved general conditions. In addition, in this preliminary study, which is a hypothesis‐generating research, we focused on dyspnea complicated by delirium in patients immediately before their death, rather than opioids for dyspnea. Further research is needed on terminal dyspnea complicated by delirium. Eighth, adverse effects were not adequately assessed. However, both opioids and antipsychotics may not have a significant impact on consciousness and communication in the natural dying process (communication capacity mildly worsened; agitated symptoms decreased; sedation levels mildly deepened, and respiratory rates were unchanged). Finally, the definition of responder (IPOS score for dyspnea ≤ 1) was not justified as a psychometric background. We are currently examining the minimal clinically important difference of IPOS for dyspnea.

## 5 Conclusions

The combined distressing symptoms of dyspnea and delirium during the last days of life are likely to comprise complex refractory suffering, which shows a poor response to pharmacological interventions, including opioids and antipsychotics. Future research needs to develop a seamless treatment strategy that will provide relief algorithms and sedatives for each symptom.

When appropriate, we suggest discussing treatment options, including sedation, with the patient and family to confirm their wishes for relief of complex suffering such as terminal dyspnea complicated by delirium.

## Author Contributions

**Takaaki Hasegawa:** study concept, statistical analysis, interpretation, and writing and editing the manuscript; **Masanori Mori:** study concept and design, data acquisition, quality control of data, interpretation, and writing and editing the manuscript; **Takashi Yamaguchi:** study concept and design, interpretation, and writing and editing the manuscript; **Kengo Imai:** study concept and design, interpretation, and writing and editing the manuscript; **Yoshinobu Matsuda:** study concept, interpretation, and writing and editing the manuscript; **Isseki Maeda:** study concept and design, interpretation, and writing and editing the manuscript; **Yutaka Hatano:** study concept and design, interpretation, and writing and editing the manuscript; **Naosuke Yokomichi:** study concept and design, interpretation, and writing and editing the manuscript; **Jun Hamano:** study concept and design, interpretation, and writing and editing the manuscript; **Tatsuya Morita:** study concept and design, interpretation, and writing and editing the manuscript.

## Conflicts of Interest

The authors declare no conflicts of interest.

## Supporting information


Data S1.

## Data Availability

The data supporting this study's results are available from the corresponding author upon reasonable request.
