# Supplementary material for: Pharmacological Strategies for Providing Patients With Delirium Relief From Terminal Dyspnea: A Secondary Data Analysis
Source: Cancer Med. 2025 Feb 11;14(3):e70677. doi: 10.1002/cam4.70677 (PMC11811727; doi:10.1002/cam4.70677)
Supplement: Supplementary file 1 — Data S1. [file CAM4-14-e70677-s001.docx]

Table S1. Distributions of dyspnea intensity and agitated symptoms

|  | All patients (n = 141) | | | | | | | |
| --- | --- | --- | --- | --- | --- | --- | --- | --- |
|  | No opioid initiation or dose escalation without antipsychotics (n = 42) | | Opioid initiation or dose escalation without antipsychotics (n = 7) | | No opioid initiation or dose escalation with antipsychotics (n = 68) | | Opioid initiation or dose escalation with antipsychotics (n = 24) | |
|  | Day 1 (%) | Day 2 (%) | Day 1 (%) | Day 2 (%) | Day 1 (%) | Day 2 (%) | Day 1 (%) | Day 2 (%) |
| IPOS worst |  |  |  |  |  |  |  |  |
| 0 | - | 3 (7) | - | 1 (14) | - | 3 (4) | - | 2 (8) |
| 1 | - | 9 (21) | - | 0 (0) | - | 10 (15) | - | 2 (8) |
| 2 | 22 (52) | 14 (33) | 4 (57) | 2 (29) | 42 (62) | 22 (32) | 15 (63) | 8 (33) |
| 3 | 17 (41) | 3 (7) | 2 (29) | 0 (0) | 22 (32) | 13 (19) | 9 (38) | 7 (29) |
| 4 | 3 (7) | 3 (7) | 1 (14) | 1 (14) | 4 (6) | 2 (3) | 0 (0) | 2 (8) |
| Cannot assess | - | 10 (24) | - | 3 (43) | - | 18 (27) | - | 3 (13) |
| MDAS item 9 |  |  |  |  |  |  |  |  |
| 0 | 19 (45) | 28 (66) | 4 (57) | 6 (86) | 23 (34) | 31 (46) | 7 (29) | 10 (42) |
| 1 | 14 (33) | 10 (24) | 2 (29)) | 1 (14) | 21 (31) | 26 (38) | 13 (54) | 9 (38) |
| 2 | 5 (12) | 3 (7) | 1 (14) | 0 (0) | 20 (29) | 9 (13) | 4 (17) | 5 (21) |
| 3 | 4 (10) | 1 (2) | 0 (0) | 0 (0) | 4 (6) | 2 (3) | 0 (0) | 0 (0) |
|  | Patients with agitated delirium (hyperactive or mixed; n = 82) | | | | | | | |
|  | No opioid initiation or dose escalation without antipsychotics  (n = 20) | | Opioid initiation or dose escalation without antipsychotics  (n = 3) | | No opioid initiation or dose escalation with antipsychotics  (n = 42) | | Opioid initiation or dose escalation  with antipsychotics  (n = 17) | |
|  | Day 1 (%) | Day 2 (%) | Day 1 (%) | Day 2 (%) | Day 1 (%) | Day 2 (%) | Day 1 (%) | Day 2 (%) |
| IPOS worst |  |  |  |  |  |  |  |  |
| 0 | - | 1 (5) | - | 0 (0) | - | 2 (5) | - | 0 (0) |
| 1 | - | 4 (20) | - | 0 (0) | - | 6 (14) | - | 1 (6) |
| 2 | 9 (45) | 7 (35) | 1 (33) | 0 (0) | 23 (55) | 9 (21) | 9 (53) | 5 (29) |
| 3 | 8 (40) | 1 (5) | 2 (66) | 0 (0) | 17 (41) | 12 (29) | 8 (47) | 7 (41) |
| 4 | 3 (15) | 2 (10) | 0 (0) | 0 (0) | 2 (5) | 1 (2) | 0 (0) | 2 (12) |
| Cannot assess | - | 5 (25) | - | 3 (100) | - | 12 (29) | - | 2 (12) |
| MDAS item 9 |  |  |  |  |  |  |  |  |
| 0 | - | 8 (40) | - | 2 (67) | - | 8 (19) | - | 3 (18) |
| 1 | 12 (60) | 9 (45) | 2 (67) | 1 (33) | 18 (43) | 23 (55) | 13 (77) | 9 (53) |
| 2 | 4 (20) | 2 (10) | 1 (33) | 0 (0) | 20 (48) | 9 (21) | 4 (24) | 5 (29) |
| 3 | 4 (20) | 1 (5) | 0 (0) | 0 (0) | 4 (10) | 2 (5) | 0 (0) | 0 (0) |
|  | Patients with hypoactive delirium (n = 59) | | | | | | | |
|  | No opioid initiation or dose escalation without antipsychotics  (n = 22) | | Opioid initiation or dose escalation without antipsychotics  (n = 4) | | No opioid initiation or dose escalation with antipsychotics  (n = 26) | | Opioid initiation or dose escalation  with antipsychotics  (n = 7) | |
|  | Day 1 (%) | Day 2 (%) | Day 1 (%) | Day 2 (%) | Day 1 (%) | Day 2 (%) | Day 1 (%) | Day 2 (%) |
| IPOS worst |  |  |  |  |  |  |  |  |
| 0 | - | 2 (9) | - | 1 (25) | - | 1 (4) | - | 2 (29) |
| 1 | - | 5 (23) | - | 0 (0) | - | 4 (15) | - | 1 (14) |
| 2 | 13 (59) | 7 (32) | 3 (75) | 2 (50) | 19 (73) | 13 (50) | 6 (86) | 3 (43) |
| 3 | 9 (41) | 2 (9) | 1 (25) | 0 (0) | 5 (19) | 1 (4) | 1 (14) | 0 (0) |
| 4 | 0 (0) | 1 (5) | 0 (0) | 1 (25) | 2 (8) | 1 (4) | 0 (0) | 0 (0) |
| Cannot assess | - | 5 (23) | - | 0 (0) | - | 6 (23) | - | 1 (14) |
| MDAS item 9 |  |  |  |  |  |  |  |  |
| 0 | 19 (86) | 20 (91) | 4 (100) | 4 (100) | 23 (89) | 23 (89) | 7 (0) | 7 (0) |
| 1 | 2 (9) | 1 (5) | 0 (0) | 0 (0) | 3 (12) | 3 (12) | 0 (0) | 0 (0) |
| 2 | 1 (5) | 1 (5) | 0 (0) | 0 (0) | 0 (0) | 0 (0) | 0 (0) | 0 (0) |
| 3 | 0 (0) | 0 (0) | 0 (0) | 0 (0) | 0 (0) | 0 (0) | 0 (0) | 0 (0) |

IPOS means Integrated Palliative Outcome Scale; MDAS means Memorial Delirium Assessment Scale.

Table S2. Proportion of responders with IPOS score for dyspnea ≤ 1

| All patients (n = 141) | | | | | | | |
| --- | --- | --- | --- | --- | --- | --- | --- |
| No opioid initiation or dose escalation without antipsychotics (n = 42) | | Opioid initiation or dose escalation without antipsychotics (n = 7) | | No opioid initiation or dose escalation with antipsychotics (n = 68) | | Opioid initiation or dose escalation with antipsychotics (n = 24) | |
| % | (95% CI) | % | (95% CI) | % | (95% CI) | % | (95% CI) |
| 29 | (17–44) | 14 | (1–53) | 19 | (11–30) | 17 | (6–37) |
| Patients with agitated delirium (hyperactive or mixed; n = 82) | | | | | | | |
| No opioid initiation or dose escalation without antipsychotics (n = 20) | | Opioid initiation or dose escalation without antipsychotics (n = 3) | | No opioid initiation or dose escalation with antipsychotics (n = 42) | | Opioid initiation or dose escalation with antipsychotics (n = 17) | |
| % | (95% CI) | % | (95% CI) | % | (95% CI) | % | (95% CI) |
| 25 | (11–47) | 0 | (0–62) | 19 | (10–34) | 6 | (0–29) |
| Patients with hypoactive delirium; n = 59) | | | | | | | |
| No opioid initiation or dose escalation without antipsychotics (n = 22) | | Opioid initiation or dose escalation without antipsychotics (n = 4) | | No opioid initiation or dose escalation with antipsychotics (n = 26) | | Opioid initiation or dose escalation with antipsychotics (n = 7) | |
| % | (95% CI) | % | (95% CI) | % | (95% CI) | % | (95% CI) |
| 32 | (16–53) | 25 | (3–71) | 19 | (8–38) | 43 | (16–75) |

IPOS means Integrated Palliative Outcome Scale; CI means confidence interval.

Missing data for IPOS at day 2 were imputed using the baseline observation carried forward method (Those with missing data were categorized as non-responders).

Table S3. Complete case analysis of changes (mean score) in Dyspnea intensity (IPOS)

| Complete case analysis among all patients (n = 107) | | | | | | | | | | | | | | | |
| --- | --- | --- | --- | --- | --- | --- | --- | --- | --- | --- | --- | --- | --- | --- | --- |
| No opioid initiation or dose escalation without antipsychotics (n = 32) | | | | Opioid initiation or dose escalation without antipsychotics (n = 4) | | | | No opioid initiation or dose escalation with antipsychotics (n = 50) | | | | Opioid initiation or dose escalation with antipsychotics (n = 21) | | | |
| Day 1  (SD) | Day 2  (SD) | Difference  (95% CI) | *P* | Day 1  (SD) | Day 2  (SD) | Difference  (95% CI) | *P* | Day 1  (SD) | Day 2  (SD) | Difference  (95% CI) | *P* | Day 1  (SD) | Day 2  (SD) | Difference  (95% CI) | *P* |
| 2.4  (0.6) | 1.8  (1.1) | 0.6 (0.2–1.0) | 0.003 | 2.5  (1.0) | 2.0  (1.6) | 0.5 (-1.1–2.1) | 0.39 | 2.5  (0.6) | 2.0  (0.9) | 0.4 (0.2–0.7) | 0.003 | 2.4  (0.5) | 2.2  (1.1) | 0.1 (-0.3–0.6) | 0.51 |
| Complete case analysis among patients with agitated delirium (hyperactive or mixed; n = 60) | | | | | | | | | | | | | | | |
| No opioid initiation or dose escalation without antipsychotics (n = 15) | | | | Opioid initiation or dose escalation without antipsychotics (n = 0) | | | | No opioid initiation or dose escalation with antipsychotics (n = 30) | | | | Opioid initiation or dose escalation with Antipsychotic (n = 15) | | | |
| Day 1  (SD) | Day 2  (SD) | Difference  (95% CI) | *P* | Day 1  (SD) | Day 2  (SD) | Difference  (95% CI) | *P* | Day 1  (SD) | Day 2  (SD) | Difference  (95% CI) | *P* | Day 1  (SD) | Day 2  (SD) | Difference  (95% CI) | *P* |
| 2.5  (0.7) | 1.9  (1.1) | 0.6 (-0.1–1.3) | 0.07 | NA | NA | NA | NA | 2.5  (0.6) | 2.1  (1.0) | 0.4 (0.0–0.8) | 0.043 | 2.5  (0.5) | 2.7  (0.8) | -0.2 (-0.6–0.2) | 0.33 |
| Complete case analysis among patients with hypoactive delirium (n = 47) | | | | | | | | | | | | | | | |
| No opioid initiation or dose escalation without antipsychotics (n = 17) | | | | Opioid initiation or dose escalation without antipsychotics (n = 4) | | | | No opioid initiation or dose escalation with antipsychotics (n = 20) | | | | Opioid initiation or dose escalation with antipsychotics (n = 6) | | | |
| Day 1  (SD) | Day 2  (SD) | Difference  (95% CI) | *P* | Day 1  (SD) | Day 2  (SD) | Difference  (95% CI) | *P* | Day 1  (SD) | Day 2  (SD) | Difference  (95% CI) | *P* | Day 1  (SD) | Day 2  (SD) | Difference  (95% CI) | *P* |
| 2.4  (0.5) | 1.7  (1.0) | 0.6 (0.1–1.2) | 0.02 | 2.0  (1.6) | 2.0  (1.6) | 0.5 (-1.1–2.1) | 0.39 | 2.4  (0.7) | 1.9  (0.8) | 0.5 (0.1–0.9) | 0.029 | 2.2  (0.4) | 1.2  (1.0) | 1.0 (0.1–1.9) | 0.04 |

IPOS means Integrated Palliative Outcome Scale; CI, confidence interval; SD, standard deviation; and NA, not available.

Table S4. Change (mean score) in Dyspnea intensity (IPOS) based on baseline observation carried forward analysis

| Baseline observation carried forward analysis among all patients (n = 141) | | | | | | | | | | | | | | | |
| --- | --- | --- | --- | --- | --- | --- | --- | --- | --- | --- | --- | --- | --- | --- | --- |
| No opioid initiation or dose escalation without antipsychotics (n = 42) | | | | Opioid initiation or dose escalation without antipsychotics (n = 7) | | | | No opioid initiation or dose escalation with antipsychotics (n = 68) | | | | Opioid initiation or dose escalation with antipsychotics (n = 24) | | | |
| Day 1  (SD) | Day 2  (SD) | Difference  (95% CI) | *P* | Day 1  (SD) | Day 2  (SD) | Difference  (95% CI) | *P* | Day 1  (SD) | Day 2  (SD) | Difference  (95% CI) | *P* | Day 1  (SD) | Day 2  (SD) | Difference  (95% CI) | *P* |
| 2.6  (0.6) | 2.1  (1.1) | 0.5 (0.2–0.8) | 0.003 | 2.6  (0.8) | 2.0  (1.6) | 0.5 (-1.1–2.1) | 0.36 | 2.4  (0.6) | 2.1  (0.9) | 0.3 (0.1–0.5) | 0.003 | 2.4  (0.5) | 2.3  (1.0) | 0.1 (-0.3–0.5) | 0.50 |
| Baseline observation carried forward analysis among patients with agitated delirium (hyperactive or mixed; n = 82) | | | | | | | | | | | | | | | |
| No opioid initiation or dose escalation without antipsychotics (n = 20) | | | | Opioid initiation or dose escalation without antipsychotics (n = 3) | | | | No opioid initiation or dose escalation with antipsychotics (n = 42) | | | | Opioid initiation or dose escalation with antipsychotics (n = 17) | | | |
| Day 1  (SD) | Day 2  (SD) | Difference  (95% CI) | *P* | Day 1  (SD) | Day 2  (SD) | Difference  (95% CI) | *P* | Day 1  (SD) | Day 2  (SD) | Difference  (95% CI) | *P* | Day 1  (SD) | Day 2  (SD) | Difference  (95% CI) | *P* |
| 2.7  (0.7) | 2.3  (1.1) | 0.5 (-0.0–0.9) | 0.07 | 2.7  (0.6) | 2.7  (0.6) | NA | NA | 2.5  (0.6)) | 2.2  (0.9) | 0.3 (0.0–0.6) | 0.044 | 2.5  (0.5) | 2.6  (0.8) | -0.2 (-0.6–0.2) | 0.33 |
| Baseline observation carried forward analysis among patients with hypoactive delirium (n = 59) | | | | | | | | | | | | | | | |
| No opioid initiation or dose escalation without antipsychotics (n = 22) | | | | Opioid initiation or dose escalation without antipsychotics (n = 4) | | | | No opioid initiation or dose escalation with antipsychotics (n = 26) | | | | Opioid initiation or dose escalation with antipsychotics (n = 7) | | | |
| Day 1  (SD) | Day 2  (SD) | Difference  (95% CI) | *P* | Day 1  (SD) | Day 2  (SD) | Difference  (95% CI) | *P* | Day 1  (SD) | Day 2  (SD) | Difference  (95% CI) | *P* | Day 1  (SD) | Day 2  (SD) | Difference  (95% CI) | *P* |
| 2.4  (0.5) | 1.9  (1.0) | 0.5 (0.1–0.9) | 0.024 | 2.5  (1.0) | 2.0  (1.6) | 0.5 (-1.1–2.1) | 0.39 | 2.4  (0.6) | 2.0  (0.8) | 0.4 (0.0–0.7) | 0.03 | 2.1  (0.4) | 1.3  (1.0) | 0.9 (0.0–1.7) | 0.045 |

IPOS means Integrated Palliative Outcome Scale; CI, confidence interval; and NA, not available.
